# Anxiety, depression, eating behaviors, and irrational food beliefs as predictors of type 2 diabetes risk in Honduran adults

**DOI:** 10.3389/fpubh.2025.1672949

**Published:** 2025-11-17

**Authors:** Estrella Maradiaga, Miguel Landa–Blanco, Raquel Mejía-Sánchez

**Affiliations:** National Autonomous University of Honduras, Tegucigalpa, Honduras

**Keywords:** type 2 diabetes mellitus, anxiety, depression, irrational food beliefs, eating behavior, health psychology

## Abstract

**Introduction:**

Chronic conditions like type 2 diabetes mellitus (T2DM) require a multidimensional understanding of health, especially in low-resource settings. This study examined the association between psychological symptoms (anxiety, depression), cognitive factors (irrational food beliefs), behavioral variables (eating patterns, physical activity), and T2DM risk in adults from Honduras.

**Methods:**

A cross-sectional survey was conducted with 336 adults using online convenience sampling. Participants completed a modified Finnish Diabetes Risk Score (FINDRISC), Patient Health Questionnaire-9 (PHQ-9), Generalized Anxiety Disorder-7 (GAD-7), Irrational Food Beliefs Scale, and the Eating Behavior Phenotype Scale. Most participants had no prior diagnosis of hypertension, elevated glucose, or diabetes, though 61.9% were overweight or obese.

**Results:**

The findings suggest that when compared to men, women reported higher symptoms of depression and emotional grazing, while also reporting lower hyperphagic eating. No significant differences were found for T2DM risk, irrational food beliefs, anxiety, hedonic eating, disorganized eating, or compulsive eating. On the other hand, anxiety, depression, and age modestly predicted T2DM risk, explaining 18.1% of its variance. Depression was linked to all eating behaviors, while irrational food beliefs predicted some eating phenotypes but not T2DM risk. Although sex was associated with specific eating patterns, it did not predict diabetes risk. Disordered eating did not mediate the relationship between psychological symptoms and T2DM risk.

**Discussion:**

The cross-sectional design used precludes causal inference, the mediation results should be interpreted as descriptive rather than causal, and the use of non-random sampling limits the generalizability of the findings. These findings highlight the importance of integrating mental health and lifestyle interventions to reduce vulnerability to type 2 diabetes, especially in at-risk populations.

## 1 Introduction

Health is a holistic state of physical, mental, and social wellbeing that integrates all constitutive dimensions of human functioning, representing a condition that enables individuals to maintain and pursue a good life (1). Yet this holistic capacity for wellbeing is increasingly threatened, as non-communicable diseases (NCDs) prevalence increases over time (2, 3), and becomes the leading cause of mortality and morbidity worldwide (4, 5). Common NCDs include cardiovascular diseases, cancer, respiratory diseases, hypertension, strokes, chronic kidney disease, and type 2 diabetes mellitus (T2DM) (6).

T2DM is a chronic condition affecting approximately 400 million individuals worldwide; it is characterized by insulin resistance and β-cell dysfunction and is frequently associated with metabolic dysfunction syndrome, which involves dyslipidemia and hyperglycemia (7, 8). T2DM is influenced by multiple risk factors, which can be categorized into genetic, lifestyle, demographic, and environmental factors (9–12).

A crucial aspect of T2DM is its association with mental health. Globally, 1 in 8 individuals has a mental disorder that affects emotional regulation, behavior, and cognition, with depression and anxiety being the most common (13, 14). Anxiety is a prevalent comorbidity in patients with T2DM and is also a risk factor for its development, significantly impacting quality of life and treatment adherence (15, 16). Similarly, numerous studies have demonstrated that depression increases the risk of T2DM by promoting insulin resistance and poor glycemic control, with a risk ratio of 1.15 for developing T2DM following a diagnosis of depression (17). However, this prevalence is bidirectional. Depression can lead to dysregulation of insulin signaling and increased inflammation, which in turn affects serotonin levels in the brain, further exacerbating depressive symptoms. This results in unhealthy behaviors and sedentary lifestyles, worsening both diabetes and depression (18, 19).

Another relevant factor in this dynamic is the individual's cognitive framework, particularly their belief system. The ABC model posits that beliefs (B) mediate the relationship between activating events (A) and emotional or behavioral responses (C) (20). In the context of food, distorted beliefs (such as “*If you exercise, it doesn't matter what you eat”*) can trigger maladaptive eating behaviors (21, 22). These irrational beliefs often contribute to unhealthy dietary patterns, including diets high in refined carbohydrates, and hinder adherence to healthier eating practices (23, 24). Moreover, such beliefs are linked to anxiety and depression, which can promote emotional eating (25).

Moreover, eating behavior refers to the complex ways in which decisions about food and eating habits are made. It is a multifaceted phenomenon determined by neurobiology, development, the environment, and an individual's health status (26). There are also different subphenotypes of eating behavior, which refer to patterns in which an individual presents attitudes toward food. These have been described in five categories: emotional grazing, hyperphagic, hedonic, disorganized, and compulsive (27). The emotional grazing eating subphenotype involves using food as a coping mechanism in response to emotions. The hyperphagic subphenotype is characterized by excessive food intake during a single eating episode. The hedonic subphenotype reflects an increased desire to eat in response to external cues or stimuli. The disorganized subphenotype describes a tendency to skip one or more main meals. Finally, the compulsive subphenotype involves rapid and excessive food consumption over a short period (27).

From the theoretical framework of health psychology, this discipline aims to minimize the potential risk of diseases while promoting healthy behaviors (28). Within this field, motivational theory posits that internal drives activate belief systems and foster new patterns of action (29). Additionally, the theory of planned behavior highlights the importance of intentions in guiding personal actions, which are shaped by attitudes, perceived social expectations, and a sense of control over one's conduct. This theory serves as a framework for promoting healthy eating habits by considering the role of attitudes and perceived control (30). Finally, the theory of reasoned action explains that individuals make decisions through a gradual process in which they rationally analyze available information and evaluate the potential consequences of their actions. Thus, behavioral rationality is based on the deliberate processing of information (31). In summary, health psychology is a multidisciplinary field that examines the interplay between psychological factors and health outcomes within the biopsychosocial model, aiming to understand behaviors related to health and illness (32).

To better understand the risk of T2DM, contextual factors must be considered. In vulnerable socioeconomic contexts such as Honduras, many individuals are unable to access quality healthcare due to the lack of resources. In 2022, Honduras reported 2.2 million people in a food crisis or emergency (33). The prevalence of overweight and obesity among individuals aged 15 years and older in Honduras in 2022 was 63.8% (34); highlighting a significant issue in public health related to nutrition. Similarly, mental health has become an increasing concern, emerging as a common public health problem (35). However, Honduras has only 0.66 psychiatrists and 0.62 psychologists per 100,000 inhabitants (36), demonstrating the limited capacity to address this issue.

**Figure 1:**
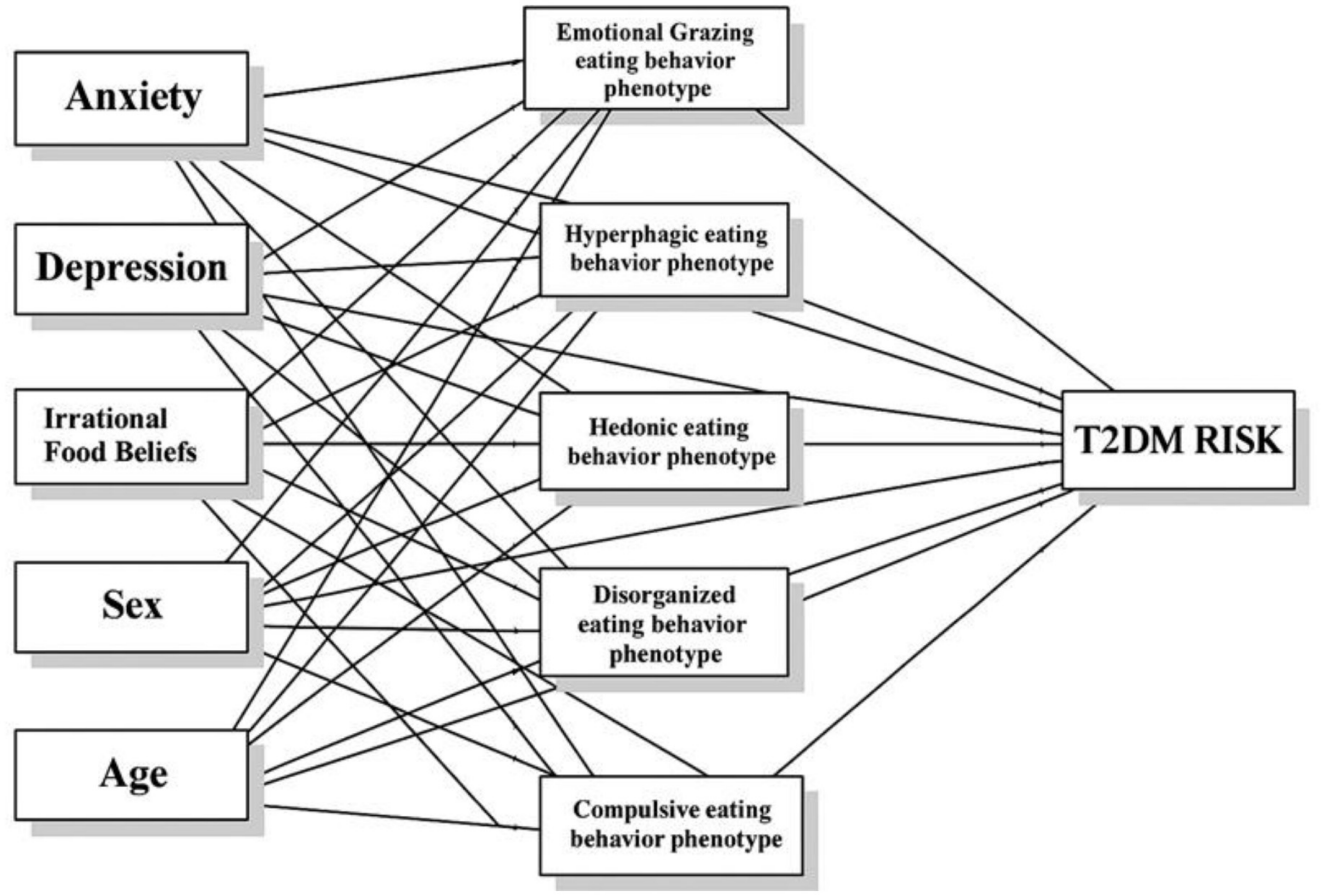
Hypothetical model.

Although T2DM is a growing public health concern in Honduras, the topic remains largely understudied. Specifically, limited empirical work has examined the psychosocial dimensions of T2DM within this context, particularly how cultural and irrational food-related beliefs shape emotional responses and eating behaviors. In Honduras, where economic constraints, food insecurity, and cultural norms strongly influence everyday life, understanding these cognitive and emotional factors is crucial. Yet, research addressing how such beliefs interact with psychological patterns to influence T2DM risk in this population remains scarce. Therefore, the purpose of this study was to investigate how psychological and demographic factors relate to T2DM risk among adults in San Pedro Sula, Honduras, with particular attention to sex differences. Specifically, the study examined whether symptoms of anxiety, depression, and irrational food beliefs, along with sex and age, are associated with distinct eating behavior phenotypes (emotional grazing, hyperphagic, hedonic, disorganized, and compulsive eating). Based on the literature and the purpose of the study, the following hypotheses were formulated:

- Hypothesis 1: Psychological factors (anxiety, depression, irrational food beliefs) and demographic variables (sex, age) are each directly associated with the eating behavior phenotype, which encompasses emotional grazing, hyperphagic, hedonic, disorganized, and compulsive eating behaviors.
- Hypothesis 2: Psychological factors (anxiety, depression, irrational food beliefs) and demographic variables (sex, age) have a direct association with T2DM risk, independent of their pathway through eating behavior phenotypes.
- Hypothesis 3: The eating behavior phenotype mediates the association between psychological and demographic factors (anxiety, depression, irrational food beliefs, sex, and age) and risk for T2DM, such that these factors are related to T2DM risk indirectly via eating behaviors (see Figure 1).

## 2 Methods

### 2.1 Participants

Participants were recruited through non-probability convenience sampling. As COVID-19 restrictions were still in place when the data collection started (April 2023), the instrument was administered online via Google Forms using a snowball sampling strategy, disseminated through social networks and interpersonal outreach. The inclusion criteria comprised: Honduran nationality, current residence in the city of San Pedro Sula, age 18 years or older, and voluntary participation through the provision of informed consent. Women with gestational diabetes were not excluded, given that this condition is recognized as a risk factor for the development of T2DM.

### 2.2 Instruments

#### 2.2.1 Modified Finnish Diabetes Risk Score (FINDRISC)

The modified Finnish Diabetes Risk Score (FINDRISC) (37) for Latin America has been used to assess an individual's risk of developing diabetes (38). This study uses a version of the FINDRISC that has been previously validated in countries like Venezuela (39). Originally, this scale consisted of eight questions related to risk factors associated with T2DM diagnosis. It measures a combination of factors, including age, body mass index (BMI), waist circumference, physical activity levels, daily consumption of fruits and vegetables, history of antihypertensive medication use, history of high blood glucose, and family history of diabetes. In our study, we excluded waist circumference from the FINDRISC assessment, as data were collected online and self-reported by participants. Given that waist circumference requires accurate measurement and may be prone to significant error when reported without guidance or standardized procedures, we determined that its inclusion could compromise the reliability of the risk estimates. Therefore, to maintain data quality and consistency, we opted to omit this variable from the final risk score calculation. The FINDRISC scoring system assigns points to several key risk factors, with higher total scores reflecting an increased likelihood of developing type 2 diabetes within the next 10 years.

#### 2.2.2 Generalized Anxiety Disorder Scale (GAD-7)

The Generalized Anxiety Disorder Scale (GAD-7) (40) was used to assess generalized anxiety; the Spanish version of the GAD-7 has been recently validated for the Honduran population (41). This instrument consists of 7 Likert-type statements. Response options include: “not at all” (0 points), “several days” (1 point), “more than half the days” (2 points), and “nearly every day” (3 points), with total scores ranging from a minimum of 0 to a maximum of 21. With the current data, the GAD-7 shows an adequate internal consistency (ω = 0.905).

#### 2.2.3 Patient Health Questionnaire-9 (PHQ-9)

The Patient Health Questionnaire-9 (PHQ-9) (42), was used to assess the frequency of symptoms related to emotional distress experienced over the past 2 weeks; the Spanish version of the PHQ-9 has been recently validated for the Honduran setting (41). The PHQ-9 is a self-report screening tool scored on a Likert scale, consisting of 9 items. Response options are “not at all” (0 points), “several days” (1 point), “more than half the days” (2 points), and “nearly every day” (3 points). The minimum possible score is 0, while the maximum is 27. In this study, the PHQ-9 demonstrated adequate internal reliability (ω = 0.917).

#### 2.2.4 Irrational Food Beliefs Scale (Spanish version)

The Spanish version of the Irrational Food Beliefs Scale (43) was used to assess irrational beliefs about food. The scale consists of 57 items, of which 41 belong to the irrational beliefs subscale and 16 to the rational beliefs subscale. Response options are “strongly disagree” (1 point), “moderately disagree” (2 points), “neither agree nor disagree” (3 points), “moderately agree” (4 points), and “strongly agree” (5 points). For this study, after recording items belonging to the rational beliefs subscale, a single total score was calculated. Higher scores indicate a higher presence of irrational food beliefs. In the current study, the questionnaire possesses adequate internal consistency (ω = 0.892).

#### 2.2.5 Eating Behavior Phenotype Scale

The Eating Behavior Phenotype Scale (EBPS) (27) is a Spanish-language self-report questionnaire developed for clinical use to identify an individual's predominant ingestive behavior subphenotype. The instrument consists of 16 items that correspond to five distinct eating behavior phenotypes: hedonic, compulsive, emotional grazing, disorganized, and hyperphagic. All rated on a scale from 1 (“never”) to 5 (“always”), specific items may be inversely recorded. A higher score indicates a higher intensity of the subphenotype. Reliability was assessed using McDonald's omega coefficient: emotional grazing subphenotype (ω = 0.854), hyperphagic subphenotype (ω = 0.652), hedonic subphenotype (ω = 0.743), disorganized subphenotype (ω = 0.658), and compulsive subphenotype (ω = 0.801).

### 2.3 Ethical considerations

Informed consent was obtained from all participants. They were provided with an explanation that their participation was part of an academic research study. Additionally, participants were informed regarding their voluntary participation and their right to access the results, which will be made publicly available in various academic settings, while ensuring the confidentiality of their identities. They were also assured that they could withdraw from the study at any time without any consequences. This research was reviewed and approved by the Research Ethics Committee of the Faculty of Social Sciences at the National Autonomous University of Honduras, with approval granted on March 31, 2023, under resolution CEIFCS-2023-P3, confirming that it meets the necessary ethical criteria for its execution.

### 2.4 Data analyses

The Strengthening the Reporting of Observational Studies in Epidemiology (STROBE) guidelines were used as a general methodological framework for the current study (44). All statistical analyses were performed using Jamovi software version 2.6.13 (45). Reliability of multi-item scales was assessed using McDonald's omega coefficient (ω). Descriptive statistics were computed for all variables, including: means (*M*), standard deviations (*SD*), minimums (MIN), and maximums (MAX).

Differences between men and women were examined using a Multivariate Analysis of Variance (MANOVA) with Pillai's Trace test. This statistic was chosen because it offers greater robustness to violations of assumptions, particularly heterogeneity of covariance matrices and non-normality (46). Then, Analysis of Variance (ANOVA) was used to detect specific statistically significant differences.

To test the hypothesized relationships, the *medmod* module was employed, specifically the *Generalized Linear Model (GLM) Mediation Model* submodule, which applies maximum likelihood estimation through multiple and conditional regression analyses. Paths were analyzed to estimate direct, indirect (mediation), and total relationships of psychological (anxiety, depression, irrational food beliefs) and demographic variables (sex, age) on T2DM risk through eating behavior phenotypes. Mediation was assessed by examining indirect pathways through individual eating behavior phenotypes. Confidence intervals for pathways were computed using the delta method. Collinearity diagnostics were based on tolerance statistics and the Variance Inflation Factor (VIF) for each predictor in the multivariate model. A *post-hoc* power analysis was calculated given the number of predictors, sample size and *R*^2^. Statistical significance was evaluated at an alpha level of *p* < 0.05. All questionnaire items were mandatory; therefore, only fully completed responses with no missing data were included in the study.

## 3 Results

### 3.1 Participants' characteristics and prevalence of health indicators

A total of 336 participants completed the survey, of whom 274 (81.5%) were women and 62 (18.4%) were men. The age of the respondents ranged from a minimum of 18 to a maximum of 71 years, with a mean of 33.5 years (*SD* = 11.6). The FINDRISC questionnaire was used to assess the risk of developing T2DM among participants. This tool evaluates a range of clinical, behavioral, and lifestyle factors, including family history of diabetes, knowledge of waist circumference, levels of physical activity, dietary habits such as the consumption of fruits, vegetables, and whole grains, prior diagnoses of hypertension or elevated blood glucose, and BMI.

Regarding family history of diabetes, 157 participants (46.7%) reported having at least one extended family member (such as grandparents, uncles, or cousins) diagnosed with diabetes. Additionally, 87 participants (25.9%) indicated a diagnosis of diabetes in their immediate family (parents, siblings, or children), while 92 participants (27.4%) reported no family history of diabetes.

Only 38.69% (*n* = 130) of participants knew their waist circumference; of this subset, 69 had measurements below the risk thresholds—less than 80 cm for women and less than 94 cm for men. Forty participants fell within an intermediate risk range (80–88 cm for women and 94–102 cm for men), and 21 participants exceeded the high-risk thresholds, with measurements above 88 cm for women and 102 cm for men. Waist circumference data are presented solely for descriptive purposes and were excluded from the FINDRISC final score, given the limited number of participants who provided self-reported measurements, which are susceptible to errors when collected online without standardized measurement procedures.

Regarding physical activity, 176 participants (52.3%) reported engaging in at least 30 min of daily exercise, whereas 160 participants (47.6%) did not meet this level of activity. In terms of dietary habits, 163 participants (48.5%) reported consuming fruits, vegetables, or whole-grain bread daily, while 173 participants (51.5%) did not consume these foods regularly.

When asked about hypertension medication, 288 participants (85.7%) reported never having been prescribed antihypertensive drugs, whereas 48 participants (14.3%) had received such prescriptions. In terms of previous detection of elevated blood glucose levels, 284 participants (84.5%) indicated no prior diagnosis, while 52 participants (15.5%) reported having been diagnosed with elevated glucose during medical check-ups.

Regarding diabetes diagnosis, 304 participants (90.5%) stated they had never been diagnosed with diabetes, 20 participants (6.0%) were unsure, 9 participants (2.7%) reported a diagnosis of type 2 diabetes mellitus, 2 participants (0.6%) reported type 1 diabetes, and 1 participant (0.3%) reported gestational diabetes.

The mean body mass index BMI was 27.47 kg/m^2^ (*SD* = 6.17), ranging from 15.48 to 52.90 kg/m^2^. Based on BMI classifications, 14 participants (4.2%) were underweight, 112 (33.3%) had normal weight, 108 (32.1 %) were overweight, and 102 participants (30.4%) were classified as obese (see Table 1).

**Table 1:** Descriptive statistics for T2DM risk indicators.

**Variable**	**Category**	***n***	**%**
Family history of diabetes	Extended family (grandparents, uncles, cousins)	157	46.7
	Immediate family (parents, siblings, children)	87	25.9
	No family history	92	27.4
Self-reported waist circumfurence^a^	Below risk threshold (< 80 cm women, < 94 cm men)	69	53.1
	Intermediate risk (80–88 cm women, 94–102 cm men)	40	30.8
	High risk (>88 cm women, >102 cm men)	21	16.2
Physical activity	≥30 min daily exercise	176	52.3
	< 30 min daily exercise	160	47.6
Dietary habits	Daily fruits, vegetables, or whole-grain bread	163	48.5
	Non-daily consumption of fruits, vegetables, or whole grain bread	173	51.5
Hypertension medication	Never prescribed	288	85.7
	Prescribed	48	14.3
Previous elevated blood glucose diagnosis	No prior diagnosis	284	84.5
	Diagnosed with elevated glucose	52	15.5
Diabetes diagnosis	Never diagnosed	304	90.5
	Unsure	20	6.0
	Type 2 diabetes mellitus	9	2.7
	Type 1 diabetes	2	0.6
	Gestational diabetes	1	0.3
Body Mass Index	Underweight (< 18.5 kg/m^2^)	14	4.2
	Normal weight (18.5–24.9 kg/m^2^)	112	33.3
	Overweight (25.0–29.9 kg/m^2^)	108	32.1
	Obese (≥30 kg/m^2^)	102	30.4

^a^Circumference descriptions were based on self-reported data from the 130 participants who knew their measurements. kg, kilograms; m^2^, squared meters.

### 3.2 Sex-based differences in T2DM risk, psychological, and behavioral measures

MANOVA was used to assess differences between men and women. Box's M-test (*p* = 0.069) indicated that the assumption of homogeneity of covariance matrices was satisfied. However, the Shapiro–Wilk test (*p* < 0.001) revealed a significant deviation from multivariate normality. Therefore, while the data met the homogeneity assumption, they violated the normality assumption. Consequently, Pillai's Trace was used for the MANOVA, as it is a robust test under these conditions, according to prior simulation studies (46).

Pillai's Trace test indicates statistically significant differences between men and women, *F*_(1, 334)_ = 5.468, *p* < 0.001. Follow-up univariate ANOVAs revealed significant sex differences in several individual measures. Women reported higher symptoms of depression (*F* = 7.60, *p* = 0.006) and emotional grazing (*F* = 6.32, *p* = 0.012); while also reporting lower hyperphagic eating (*F* = 7.40, *p* = 0.007) compared to men. No significant differences were found for irrational food beliefs, anxiety, hedonic eating, disorganized eating, or compulsive eating (*p* > 0.05). An important consideration is that women reported higher T2DM risk scores (*F* = 3.88, *p* = 0.0498). However, this *p-value* (0.0498) was considered marginal and interpreted with caution, representing a suggestive rather than conclusive difference. See Table 2 for further details.

**Table 2:** Descriptive statistics of the variables by sex.

**Variable**	**Men**				**Women**				**Difference**	
	**Mean**	**SD**	**Min**	**Max**	**Mean**	**SD**	**Min**	**Max**	***F***	***p***
T2DM risk	7.57	3.69	2	18	8.55	3.53	0	18	3.876	0.049^a^
Irrational food beliefs	106.45	16.4	82	142	109.37	17.19	73	188	0.846	0.358
Anxiety	6.77	5.34	0	20	9.33	5.66	0	21	10.498	0.358
Depression	7.27	6.39	0	25	9.99	7.13	0	27	7.603	0.006
Emotional grazing	7.71	3.22	4	19	9.08	4.01	4	20	6.316	0.012
Hyperphagic eating	7.08	2.58	3	14	6.23	2.14	3	14	7.402	0.007
Hedonic eating	11.4	3.4	4	19	11.14	3.82	4	20	0.245	0.621
Disorganized eating	9.11	1.99	5	13	8.84	2.15	3	15	0.866	0.353
Compulsive eating	5.37	2.34	2	10	4.79	2.19	2	10	3.440	0.065

T2DM, Type 2 Diabetes Mellitus. Multivariate differences were assessed using MANOVA, followed by separate ANOVAs for each measure (F). Significant p-values are presented in bold. ^a^Exact p-value= 0.0498; this significance is marginal and should be interpreted with caution, indicating a suggestive rather than definitive difference that requires confirmation in future research.

### 3.3 Variables related to T2DM risk

The analysis of the coefficient of determination (*R*^2^) for the overall model predicting T2DM risk indicates that the predictor variables—anxiety, depression, irrational food beliefs, age, and sex—account for 18.1% of the total variance in T2DM risk (*R*^2^ = 0.181). Given the number of predictors, the sample size, and the observed *R*^2^, *post-hoc* power analysis indicated a statistical power of approximately 0.999 at the α = 0.05 level, demonstrating that the model had sufficient power to detect the observed effect size. However, this represents a moderate level of predictive power within the context of public health and risk-related behaviors. Additionally, the model was statistically significant (*p* < 0.001), suggesting that these variables contribute to the prediction of T2DM risk (see Table 2). The collinearity diagnostics suggest that multicollinearity is not a concern in the model, as the variance inflation factor (VIF = 1.087–3.153) and tolerance values (0.317–0.920) fall within acceptable thresholds.

Specifically, the emotional grazing eating behavior subphenotype exhibited the highest proportion of explained variance (*R*^2^ = 0.356, *p* < 0.001), followed by the hyperphagic (*R*^2^ = 0.218, *p* < 0.001), hedonic (*R*^2^ = 0.179, *p* < 0.001), and compulsive (*R*^2^ = 0.168, *p* < 0.001) eating behavior subphenotypes. These results indicate that the predictors selected for this study are statistically significantly associated with these specific eating behavior subphenotypes. Although significant, the disorganized eating behavior phenotype showed a considerably lower proportion of explained variance (*R*^2^ = 0.041, *p* = 0.016), suggesting that this model accounts for only a small portion of its variability.

Additionally, the model reveals that symptoms of anxiety are directly related to the risk of T2DM (β = 0.227, *p* = 0.006), indicating that higher anxiety levels are associated with increased risk. In contrast, depression does not significantly predict T2DM risk through a direct path (*p* = 0.340); however, its total relationship, which includes indirect pathways via eating behaviors, is significant (β = 0.190, *p* = 0.022).

Depressive symptoms are strongly associated with increases across all measured eating behaviors, including emotional grazing, hyperphagic, hedonic, and compulsive eating subphenotypes (*p* < 0.001). At a more granular level, they are also linked to emotional grazing, hyperphagic, disorganized, and compulsive patterns (*p* < 0.05). Similarly, irrational beliefs about food significantly predict emotional grazing (β = 0.216, *p* < 0.001) and hyperphagic eating (β = 0.154, *p* = 0.003). However, they do not directly (*p* = 0.648) or indirectly (*p* = 0.159) predict T2DM risk.

Sex is related to eating behaviors, with men showing lower levels of hyperphagic (β = −0.204, *p* < 0.001) and compulsive eating (β = −0.167, *p* < 0.001). Despite these associations, sex does not have a significant total relationship with T2DM risk (*p* = 0.514). Age, on the other hand, emerges as a robust predictor. It has a significant direct relationship with T2DM risk (β = 0.235, *p* < 0.001) and is positively associated with compulsive eating (β = 0.125, *p* = 0.019).

In summary, total pathway analyses show that T2DM risk is significantly predicted by symptoms of anxiety (*p* = 0.005), depression (*p* = 0.022), and age (*p* < 0.001). In contrast, neither irrational food beliefs nor sex significantly predicts T2DM risk. Furthermore, indirect pathway analyses indicate that none of the variables—anxiety, depression, irrational food beliefs, sex, or age—predict T2DM risk through statistically significant mediating pathways, see Table 3.

**Table 3:** Mediation analysis.

**Type**	**Pathway**	**Estimate**	**SE**	**Lower**	**Upper**	**β**	***z***	***p***
Indirect^a^ (A → B → T2DM Risk)	Anxiety → Emotional grazing →	0.009	0.008	−0.007	0.025	0.015	1.106	0.269
	Anxiety → Hyperphagic →	−0.006	0.007	−0.020	0.008	−0.010	−0.876	0.381
	Anxiety → Hedonic →	0.001	0.005	−0.009	0.011	0.001	0.175	0.861
	Anxiety → Disorganized →	=0	0.002	−0.003	0.003	=0	0.024	0.981
	Anxiety → Compulsive →	=0	0.002	−0.004	0.003	=0	−0.149	0.882
	Depression → Emotional grazing →	0.028	0.017	−0.004	0.061	0.056	1.698	0.089
	Depression → Hyperphagic →	0.025	0.014	−0.003	0.053	0.05	1.780	0.075
	Depression → Hedonic →	0.002	0.013	−0.023	0.028	0.005	0.177	0.860
	Depression → Disorganized →	=0	0.005	−0.009	0.009	=0	0.024	0.981
	Depression → Compulsive →	−0.002	0.011	−0.024	0.020	−0.003	−0.155	0.877
	IBF → Emotional grazing →	0.006	0.004	−0.001	0.014	0.030	1.670	0.095
	IBF → Hyperphagic →	0.004	0.002	−0.001	0.009	0.019	1.609	0.108
	IBF → Hedonic →	=0	0.001	−0.002	0.002	0.001	0.175	0.861
	IBF → Disorganized →	=0	0.001	−0.002	0.002	=0	−0.024	0.981
	IBF → Compulsive →	=0	0.001	−0.003	0.002	−0.001	−0.154	0.877
	Sex → Emotional grazing →	0.057	0.066	−0.072	0.187	0.006	0.870	0.384
	Sex → Hyperphagic →	−0.233	0.134	−0.496	0.031	−0.025	−1.731	0.084
	Sex → Hedonic →	−0.013	0.071	−0.152	0.127	−0.001	−0.176	0.860
	Sex → Disorganized →	−0.001	0.038	−0.076	0.074	=0	−0.024	0.981
	Sex → Compulsive →	0.015	0.098	−0.177	0.208	0.002	0.155	0.877
	Age → Emotional grazing →	=0	0.002	−0.004	0.004	=0	0.023	0.982
	Age → Hyperphagic →	−0.003	0.003	−0.009	0.002	−0.011	−1.271	0.204
	Age → Hedonic →	=0	0.001	−0.001	0.002	=0	0.169	0.866
	Age → Disorganized →	=0	0.001	−0.001	0.001	=0	0.024	0.981
	Age → Compulsive →	=0	0.002	−0.005	0.004	−0.001	−0.155	0.877
Component	Anxiety → Emotional grazing	0.071	0.050	−0.028	0.170	0.103	1.407	0.159
	Emotional Grazing → Risk	0.129	0.072	−0.012	0.270	0.141	1.788	0.074
	Anxiety → Hyperphagic	−0.032	0.032	−0.094	0.031	−0.080	−0.986	0.324
	Hyperphagic → Risk	0.197	0.104	−0.006	0.401	0.124	1.904	0.057
	Anxiety → Hedonic	0.066	0.055	−0.041	0.173	0.100	1.204	0.229
	Hedonic → Risk	0.013	0.075	−0.134	0.161	0.014	0.177	0.860
	Anxiety → Disorganized	0.018	0.033	−0.048	0.083	0.048	0.535	0.593
	Disorganized → Risk	0.002	0.087	−0.169	0.173	0.001	0.024	0.981
	Anxiety → Compulsive	0.018	0.033	−0.046	0.082	0.045	0.541	0.589
	Compulsive → Risk	−0.016	0.103	−0.217	0.185	−0.01	−0.155	0.877
	Depression → Emotional grazing	0.220	0.041	0.141	0.300	0.399	5.427	< 0.001
	Depression → Hyperphagic	0.129	0.026	0.079	0.179	0.406	5.014	< 0.001
	Depression → Hedonic	0.175	0.044	0.089	0.261	0.330	3.981	< 0.001
	Depression → Disorganized	0.054	0.027	0.001	0.106	0.179	1.999	0.046
	Depression → Compulsive	0.108	0.026	0.057	0.160	0.343	4.113	< 0.001
	IBF → Emotional grazing	0.050	0.011	0.029	0.070	0.216	4.670	< 0.001
	IBF → Hyperphagic	0.020	0.007	0.007	0.033	0.154	3.006	0.003
	IBF → Hedonic	0.013	0.011	−0.010	0.035	0.059	1.132	0.258
	IBF → Disorganized	−0.010	0.007	−0.024	0.004	−0.080	−1.416	0.157
	IBF → Compulsive	0.012	0.007	−0.001	0.026	0.096	1.816	0.069
	Sex → Emotional grazing	0.447	0.448	−0.432	1.325	0.044	0.996	0.319
	Sex → Hyperphagic	−1.178	0.284	−1.734	−0.622	−0.204	−4.151	< 0.001
	Sex → Hedonic	−0.942	0.485	−1.892	0.008	−0.098	−1.943	0.052
	Sex *ightarrow* Disorganized	−0.440	0.296	−1.021	0.141	−0.081	−1.485	0.138
	Sex → Compulsive	−0.956	0.290	−1.525	−0.387	−0.167	−3.292	< 0.001
	Age → Emotional grazing	=0	0.016	−0.030	0.031	0.001	0.023	0.982
	Age → Hyperphagic	−0.017	0.01	−0.036	0.003	−0.088	−1.708	0.088
	Age → Hedonic	0.010	0.017	−0.023	0.043	0.031	0.586	0.558
	Age → Disorganized	0.008	0.010	−0.013	0.028	0.042	0.737	0.461
	Age → Compulsive eating	0.024	0.010	0.004	0.044	0.125	2.353	0.019
Direct	Anxiety	0.143	0.051	0.042	0.243	0.227	2.776	0.006
	Depression	0.041	0.043	−0.044	0.127	0.082	0.954	0.340
	IBF	0.005	0.011	−0.017	0.027	0.024	0.457	0.648
	Sex	0.476	0.475	−0.455	1.407	0.052	1.002	0.317
	Age	0.072	0.016	0.040	0.104	0.235	4.463	< 0.001
Total (direct + indirect)	Anxiety	0.146	0.052	0.044	0.249	0.232	2.802	0.005
	Depression	0.096	0.042	0.014	0.178	0.19	2.286	0.022
	IBF	0.015	0.011	−0.006	0.037	0.074	1.409	0.159
	Sex	0.302	0.464	−0.606	1.211	0.033	0.652	0.514
	Age	0.069	0.016	0.037	0.100	0.223	4.228	< 0.001

T2DM, Type 2 Diabetes Mellitus; IBF, Irrational Beliefs about Food. The table presents the output of the GLM Mediation Model; 95% confidence intervals were computed via the standard (delta) method. Significant p-values (< 0.05) are presented in bold. ^a^T2DM Risk is the outcome for all indirect pathways.

## 4 Discussion

The present study provides empirical support for the central role of psychological processes in shaping vulnerability to T2DM in socioeconomically disadvantaged contexts. Guided by a health psychology framework, the findings highlight anxiety and depression not merely as comorbidities, but as integral components of behavioral risk profiles associated with chronic disease. This study showed that most participants had family members with diabetes; however, few were aware of key indicators such as waist circumference. Although dietary and physical activity habits were moderate, a significant portion exhibited overweight, highlighting the urgent need to promote healthy and active lifestyles to prevent chronic diseases in this population. Among the psychological variables studied, anxiety symptoms showed the strongest direct association with increased T2DM risk. This relationship persisted independently of eating behavior patterns, suggesting that the relationship between anxiety and T2DM risk may operate through generalized behavioral dysregulation, impaired decision-making, or a diminished capacity for consistent self-care (47).

For clinicians, this finding reinforces the need to treat anxiety not only as a distressing emotional state but as a behavioral vulnerability factor that may compromise the adoption or maintenance of health-promoting habits. Anxiety often interferes with routine planning (48), reduces perceived behavioral control (49), and fosters avoidance (50), effects that may be particularly pronounced in Honduras. In this setting, exposure to violent, unsafe, and socioeconomically unstable environments contributes to excessive worry and elevated anxiety prevalence (35, 51).

In contrast, the relationship between depressive symptoms and T2DM risk appeared to be more behaviorally mediated. Although depression was not a directly related factor, the total pathway was statistically significant. Participants reporting more depressive symptoms were significantly more likely to endorse emotional, compulsive, and hyperphagic eating phenotypes—styles of eating characterized by diminished self-regulation (52), emotional reactivity (53), and disengagement from internal cues of hunger and satiety (54). These patterns are consistent with models of experiential avoidance and reinforcement-based frameworks, in which food serves as a readily available strategy for emotion regulation (55). Importantly, these eating styles trended toward increasing T2DM risk, supporting the idea that depressive symptoms contribute to risk not in isolation but by altering behavior in ways that accumulate harm over time. This underscores the value of targeting emotional awareness (56), distress tolerance (57), and behavior activation within T2DM prevention programs (58), particularly for individuals with subclinical depressive symptoms who may otherwise go undetected in primary care settings.

Irrational beliefs about food, while predictive of certain eating phenotypes, did not demonstrate a significant direct or total relationship with T2DM risk. This finding departs from expectations based on cognitive models of health behavior (58), and -speculatively- may indicate that maladaptive cognitions require activation by emotional states such as anxiety or depression, to translate into behavior that meaningfully impacts health. It also raises the possibility that cognitive beliefs, in the absence of emotional dysregulation or environmental triggers, may remain dormant or be partially counteracted by competing beliefs and habits. For clinical practice, this suggests that interventions targeting beliefs alone, without addressing affective states or behavioral reinforcement patterns, may be insufficient to alter disease risk in this population meaningfully. However, further research is needed to clarify this.

Not all eating phenotypes were equally predictive of T2DM risk. Emotional grazing and hyperphagic eating displayed the most consistent associations with both depressive symptoms and elevated risk scores, suggesting that these styles may serve as relevant associated factors that suggest a possible link between emotional distress and chronic illness. These subphenotypes reflect difficulties with impulse control (59), affective modulation, and behavioral consistency—core targets in evidence-based psychological interventions such as dialectical behavior therapy (DBT), mindfulness-based interventions, and acceptance and commitment therapy (ACT) (49, 60). In contrast, hedonic, disorganized, and compulsive eating showed weaker or non-significant relationships with risk, which may reflect their more contextual or transient nature. These distinctions highlight the importance of evaluating eating patterns not as a single construct, but as differentiated psychological profiles with varying degrees of relevance to health outcomes.

Sociodemographic variables played a secondary but clarifying role. Age was positively associated with T2DM risk, as expected, but also showed an association with compulsive eating, possibly reflecting cumulative behavioral patterns across the lifespan. Gender differences emerged in the expression of some eating behaviors—women reported more emotional grazing, while men exhibited higher levels of hyperphagic behaviors. These findings are partially consistent with previous studies that suggest that interventions may benefit from considering a gender-based approach (61).

Taken together, these findings provide modest support for integrating psychological theory and intervention within chronic disease prevention. Health behaviors do not exist in isolation; they are shaped and sustained by affective states, cognitive frameworks, and behavioral repertoires. When these systems become dysregulated, they set the stage for sustained patterns of behavior that undermine health. For individuals at risk of T2DM, psychological assessment should not be an optional add-on but a core component of prevention. Interventions should not only educate about healthy lifestyles but should also address the emotional and cognitive barriers that prevent individuals from enacting those behaviors consistently. In settings with limited resources, brief, scalable interventions that target emotion regulation and eating behavior may offer a realistic and impactful alternative to traditional models focused solely on diet and physical activity.

This research is relevant beyond the Honduran context, extending to other LMICs where the multifactorial nature of T2DM includes strong psychological components. Mental health factors are closely linked to disease risk and management. In resource-limited settings with social and economic challenges, strengthening healthcare workers' psychoeducational skills and community engagement is essential to improve distress recognition, coping, and motivation. These actions should incorporate culturally adapted self-care strategies and align with health system and policy initiatives to enhance overall wellbeing (62, 63). In Honduras, where access to mental health care is restricted and poverty and low educational attainment are prevalent, evidence-based policies must address both social and psychological determinants of health. The Ministry of Health (SESAL) can build on the primary healthcare system and community networks to train staff and health promoters to identify anxiety, depression, and diabetes risk. Using standardized instruments such as the GAD-7, PHQ-9, and FINDRISC can support this effort.

Additionally, collaboration with the National Autonomous University of Honduras (UNAH) can support the development of integrated programs that connect mental health, nutrition, and chronic disease prevention, ensuring that interventions remain both evidence-based and culturally relevant. Community partners, including churches, schools, and neighborhood associations (“patronatos”), can play a key role in delivering education on food beliefs, emotional eating, and healthier preparation of traditional foods. Public communication channels like local radio and WhatsApp can further promote low-cost physical activity and balanced diets. Integrating mental health support into SESAL's chronic disease programs would strengthen prevention and care for rural and low-income populations. This approach addresses barriers to medical and psychological services and enhances overall health outcomes. The model could also inform effective, sustainable interventions in Honduras and comparable LMICs.

Despite the value of the findings, several important limitations must be acknowledged. First, the cross-sectional design prevents causal interpretation; the temporal ordering between psychological symptoms, behavioral factors, mediators, and T2DM risk cannot be established, making reverse causality possible. The mediation analyses, while assessing direct, indirect, and total associations, are descriptive rather than causal. The model explained 18.1% of the variance in T2DM risk, a modest proportion that should not be interpreted as strong predictive power at the individual level. Second, the reliance on self-reported data may have introduced recall and reporting biases. Third, T2DM risk was measured as a composite score rather than through individual risk components, limiting the ability to determine which specific factors, such as BMI, blood pressure, or family history, are most closely linked to psychological and behavioral predictors. Fourth, the FINDRISC tool was modified to exclude waist circumference, a key indicator of central adiposity, reducing comparability with studies using the standard version and limiting external validity. Fifth, the classification of eating behavior phenotypes did not account for differences in dietary quality. For example, a participant whose atypical eating involves fruits and vegetables would be grouped with one who consumes snacks or calorie-dense foods. This approach may obscure meaningful distinctions in nutritional quality and metabolic relevance within behavioral categories. Finally, sampling choices limit generalizability. Non-random recruitment through convenience and snowball methods, adopted during COVID-19 restrictions, raises concerns about representativeness and potential sampling bias. Self-selection into an online survey may also introduce information bias if participants differ systematically from non-participants in mental health status, health literacy, or motivation to report symptoms. Limited internet access could further underrepresent groups at greater psychosocial or health risk. These design, measurement, and sampling limitations should be considered when interpreting the results.

Future research using prospective designs is needed to clarify temporal ordering and to evaluate targeted psychological interventions for individuals at elevated risk. Additional work should examine contextual moderators such as social support, trauma exposure, and access to food, which may influence the manifestation of psychological risk patterns. Because the current study focused on T2DM risk as a composite variable, future studies should analyze specific risk factors such as BMI, blood pressure, and fasting glucose to better identify the pathways linking psychological and behavioral variables to metabolic outcomes. Given the cross-sectional design, longitudinal, and mixed-method approaches would strengthen causal inference and provide a deeper understanding of these mechanisms. Future studies with larger samples and latent variable modeling aims could extend this work using SEM to validate the proposed hypothetical structure. Methodological improvements, including representative sampling and the use of objective health measures, are also needed to reduce bias and enhance generalizability. Finally, considering the structural barriers to healthcare access in this region, scalable and context-sensitive interventions such as community-based psychoeducation, digital mental health tools, and integration of psychological screening into primary care should remain a research and policy priority.

In sum, this study reinforces that psychological distress is not ancillary to physical health; it is foundational. In the context of T2DM risk, anxiety and depression are not merely co-occurring problems but central elements of the risk architecture. Effective prevention must consider the ways people think, feel, and cope under stress. Behavioral interventions that fail to address this complexity risk falling short.

## Data Availability

The raw data supporting the conclusions of this article will be made available by the authors, without undue reservation.
